# XG-ac4C: identification of N4-acetylcytidine (ac4C) in mRNA using eXtreme gradient boosting with electron-ion interaction pseudopotentials

**DOI:** 10.1038/s41598-020-77824-2

**Published:** 2020-12-01

**Authors:** Waleed Alam, Hilal Tayara, Kil To Chong

**Affiliations:** 1 Department of Electronics and Information Engineering, Jeonbuk National University, Jeonju, 54896 South Korea; 2 School of International Engineering and Science, Jeonbuk National University, Jeonju, 54896 South Korea; 3 Advanced Electronics and Information Research Center, Jeonju, 54896 South Korea

**Keywords:** Computational biology and bioinformatics, Genetics, Engineering

## Abstract

N4-acetylcytidine (ac4C) is a post-transcriptional modification in mRNA which plays a major role in the stability and regulation of mRNA translation. The working mechanism of ac4C modification in mRNA is still unclear and traditional laboratory experiments are time-consuming and expensive. Therefore, we propose an XG-ac4C machine learning model based on the eXtreme Gradient Boost classifier for the identification of ac4C sites. The XG-ac4C model uses a combination of electron-ion interaction pseudopotentials and electron-ion interaction pseudopotentials of trinucleotide of the nucleotides in ac4C sites. Moreover, Shapley additive explanations and local interpretable model-agnostic explanations are applied to understand the importance of features and their contribution to the final prediction outcome. The obtained results demonstrate that XG-ac4C outperforms existing state-of-the-art methods. In more detail, the proposed model improves the area under the precision-recall curve by 9.4% and 9.6% in cross-validation and independent tests, respectively. Finally, a user-friendly web server based on the proposed model for ac4C site identification is made freely available at http://nsclbio.jbnu.ac.kr/tools/xgac4c/.

## Introduction

More than 160 different RNA modifications have been identified^1^. Among them, N4-acetylcytidine (ac4C) has regulatory potential. It occurs on cytidine and it is the only acetylation modification in eukaryotic mRNA^2^. The role of ac4C in the regulation of mRNA translation and promotion of translation efficiency was established by Arango et al.^3^ An analysis of the half-life of mRNA showed that the acetylation level and stability of target mRNA are positively correlated. Also, ac4C enhances translation when presented within the wobble sites of cytidine^3^. Furthermore, ac4C is co-related with the progression, prognosis, and development of several human diseases^4^.

Recently, Arango et al.^3^ reported that NAT10 acetyltransferase is involved in the catalyzation of N4-acetyl-cytidine (ac4C) as an mRNA modification^5^. Whole transcriptome mapping of ac4C reveals abundantly acetylated regions within the coding sequence. NAT10 mutation decreases detection of ac4C at the mapped mRNA site and is associated with down-regulation of target mRNA. So, the acetylated residues expand the repertoire of mRNA modifications to establish the role of ac4C in the regulation of mRNA translation.

**Figure 1:**
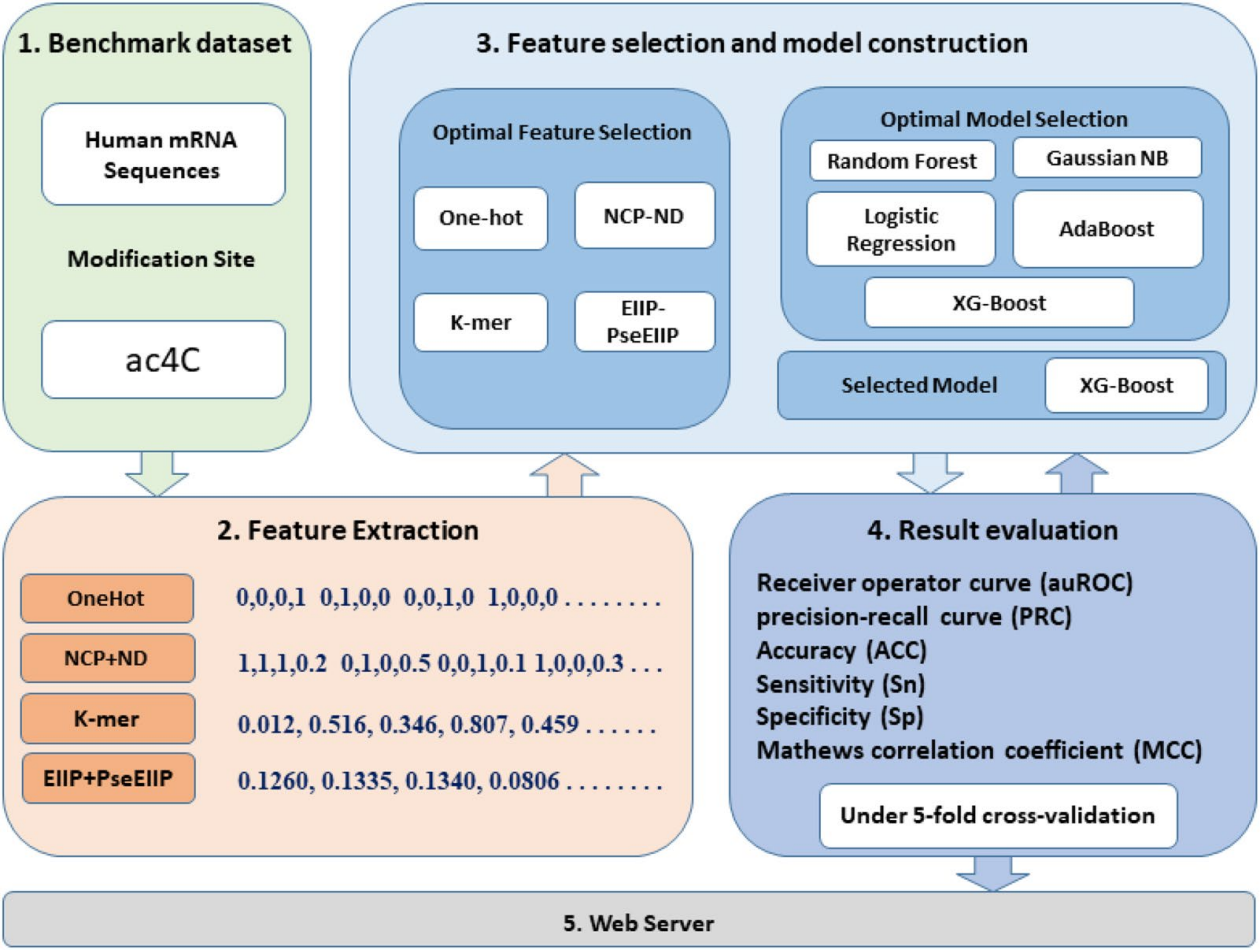
Illustration of the proposed model XG-ac4C.

More recently, the PACES predictor was proposed for classification of the ac4C modification sites in human mRNA^6^. PACES combines two random forest classifiers, position-specific di-nucleotide sequence profiles and K-nucleotide frequencies. The results of PACES can be further improved upon. Therefore, in this study, we propose a computational model based on the eXtreme Gradient Boosting (XGboost) method to identify ac4C modification sites in mRNA. The nucleotide chemical property (NCP), nucleotide density (DN), Kmer, one-hot encoding, electron-ion interaction pseudopotentials (EIIP), and electron-ion interaction pseudopotentials of trinucleotide (PseEIIP) were utilized to represent mRNA sequences in the benchmark datasets. We employed various evaluation metrics to assess XG-ac4C, all of which are commonly used in the field of bioinformatics^7–11^, namely, accuracy, sensitivity, specificity, and Matthews correlation coefficient. Furthermore, we applied 5-fold cross-validation with evaluation metrics to evaluate XG-ac4C. We also focus on the receiver operating characteristic curve (ROC) and the precision-recall curve (PRC) because the datasets are imbalanced^12^. Therefore, the optimal features representation vector and the optimal machine learning classifier are selected based on the ROC and PRC performance. The proposed model XG-ac4C is illustrated in Fig. 1. Moreover, we built a user-friendly web server for the proposed model, which is freely accessible at http://nsclbio.jbnu.ac.kr/tools/xgac4c/.

## Results and discussion

In this section, we discuss the results and the comparison with other machine learning classifiers and state-of-the-art methods. Finally, we discuss the importance of features for the XGboost classifier.

### Comparison with other machine learning classifiers

**Figure 2:**
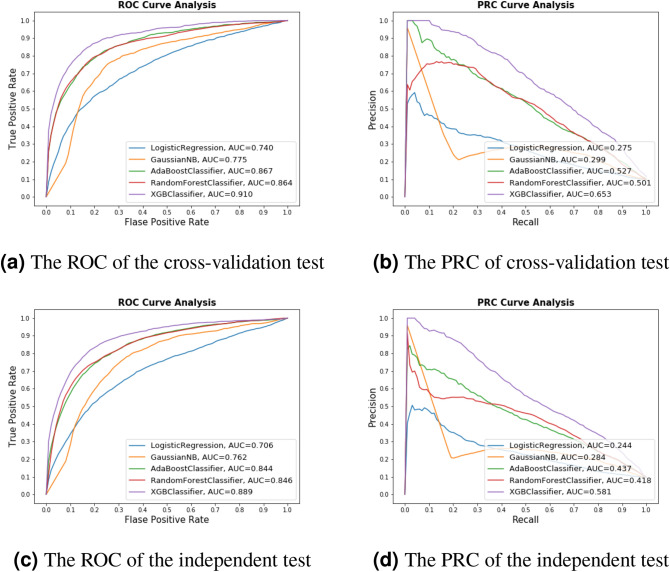
The ROC and PRC of the proposed model on the cross-validation and independent test datasets.

We tested XGboost with different feature representations, namely, one-hot, a combination of NCP and ND, k-mer, and a combination of EIIP and PseEIIP. The cross-validation test results show that the XGboost classifier with the combination of EIIP and PseEIIP outperforms instead of the other classifiers and feature representation techniques, as shown in Table 1. Therefore, we adopt the combination of EIIP and PseEIIP to encode mRNA sequences for ac4C site identification. Furthermore, we tested different machine learning algorithms, such as eXtreme Gradient Boosting (XGboost), random forest^13^, AdaBoost^14^, GaussianNB^15^, and logistic regression^16^. XGboost outperforms the aforementioned machine learning algorithms. Figure 2 shows the ROC and PRC of XGboost and the other machine learning algorithms using the combination of EIIP and PseEIIP. Moreover, the ROC and PRC of 5-fold cross-validation for all feature representation are shown in Supplementary Figure 1. It is also evident that the XGboost classifier significantly outperforms the other machine learning algorithms in terms of ROC and PRC.

**Table 1:** A comparison of the cross-validation performance between XGboost and other machine learning algorithms using different feature representations.

Classifiers	Feature	ACC	SP	SN	MCC	ROC	PRC
Logistic regression	one-hot	0.887	0.939	0.393	0.340	0.801	0.395
	NCP-ND	0.885	0.939	0.387	0.332	0.796	0.376
	K-mer	0.903	0.991	0.081	0.172	0.849	0.415
	EIIP-PseEIIP	0.903	0.998	0.007	0.046	0.740	0.275
GaussianNB	one-hot	0.792	0.806	0.668	0.328	0.810	0.352
	NCP-ND	0.737	0.759	0.526	0.191	0.732	0.327
	K-mer	0.748	0.749	0.741	0.317	0.807	0.368
	EIIP-PseEIIP	0.823	0.853	0.537	0.298	0.775	0.299
AdaBoost	one-hot	0.900	0.975	0.205	0.266	0.784	0.369
	NCP-ND	0.903	0.974	0.238	0.299	0.822	0.380
	K-mer	0.907	0.974	0.279	0.342	0.848	0.421
	EIIP-PseEIIP	0.918	0.976	0.369	0.441	0.867	0.527
Random forest	one-hot	0.902	0.998	0.007	0.034	0.772	0.370
	NCP-ND	0.904	0.997	0.033	0.121	0.798	0.349
	K-mer	0.917	0.987	0.261	0.394	0.871	0.506
	EIIP-PseEIIP	0.907	0.997	0.069	0.205	0.864	0.501
XGboost	one-hot	0.921	0.981	0.361	0.458	0.871	0.572
	NCP-ND	0.924	0.973	0.467	0.511	0.884	0.595
	K-mer	0.887	0.918	0.601	0.453	0.877	0.522
	EIIP-PseEIIP	0.921	0.956	0.597	0.552	0.910	0.653

### Comparison with the existing method

**Figure 3:**
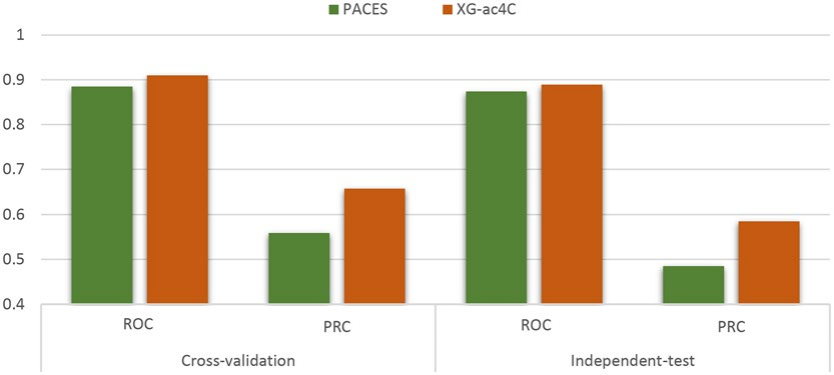
A comparison between the proposed model, XG-ac4C, and the existing model, PACES, based on ROC and PRC.

To further demonstrate the superiority of the XG-ac4C model, we compared it with a previously developed method, PACES^6^. In this study, to enable a fair comparison, we utilized the same imbalanced datasets with positive and negative samples in a ratio of 1:9. The 5-fold cross-validation and independent test set results of XG-ac4C and PACES are shown in Table 2 and Fig. 3. Since the training and independent datasets are imbalanced, the PRC is the most important parameter to compare the performance of the two methods^12^. XG-ac4C improves PRC by 9.4% and 9.6% on the cross-validation and independent test, respectively.

**Table 2:** A comparison of the performance of the proposed model, XG-ac4C, with the existing computational model PACES.

Dataset	Method	ROC	PRC
Cross-validation	PACES	0.885	0.559
	XG-ac4C	0.91	0.653
Indenpendent-test	PACES	0.874	0.485
	XG-ac4C	0.889	0.581

### Feature importance and their contribution

**Figure 4:**
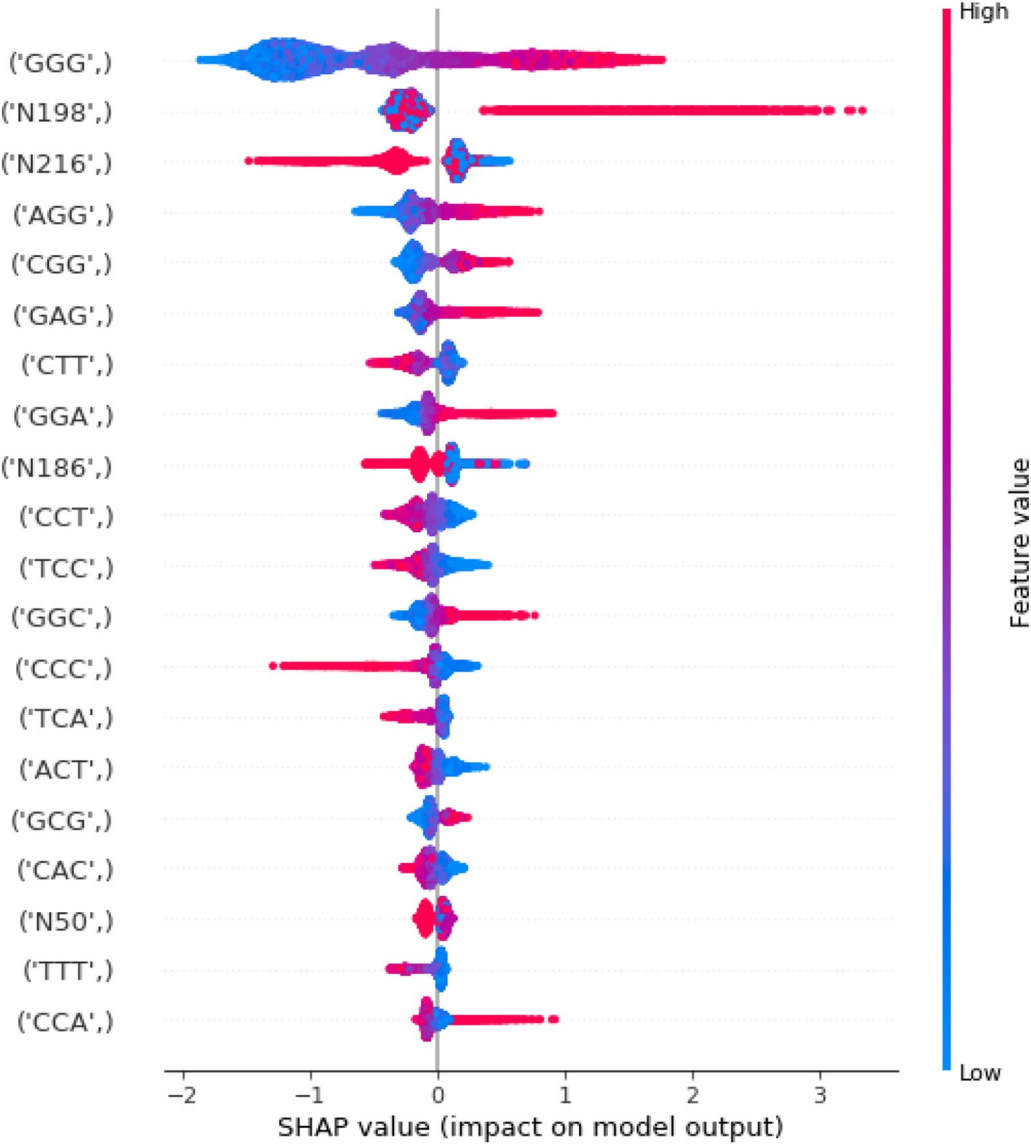
A summary of SHAP values, representing the top 20 most important features for training of the proposed model for ac4C site classification.

**Figure 5:**
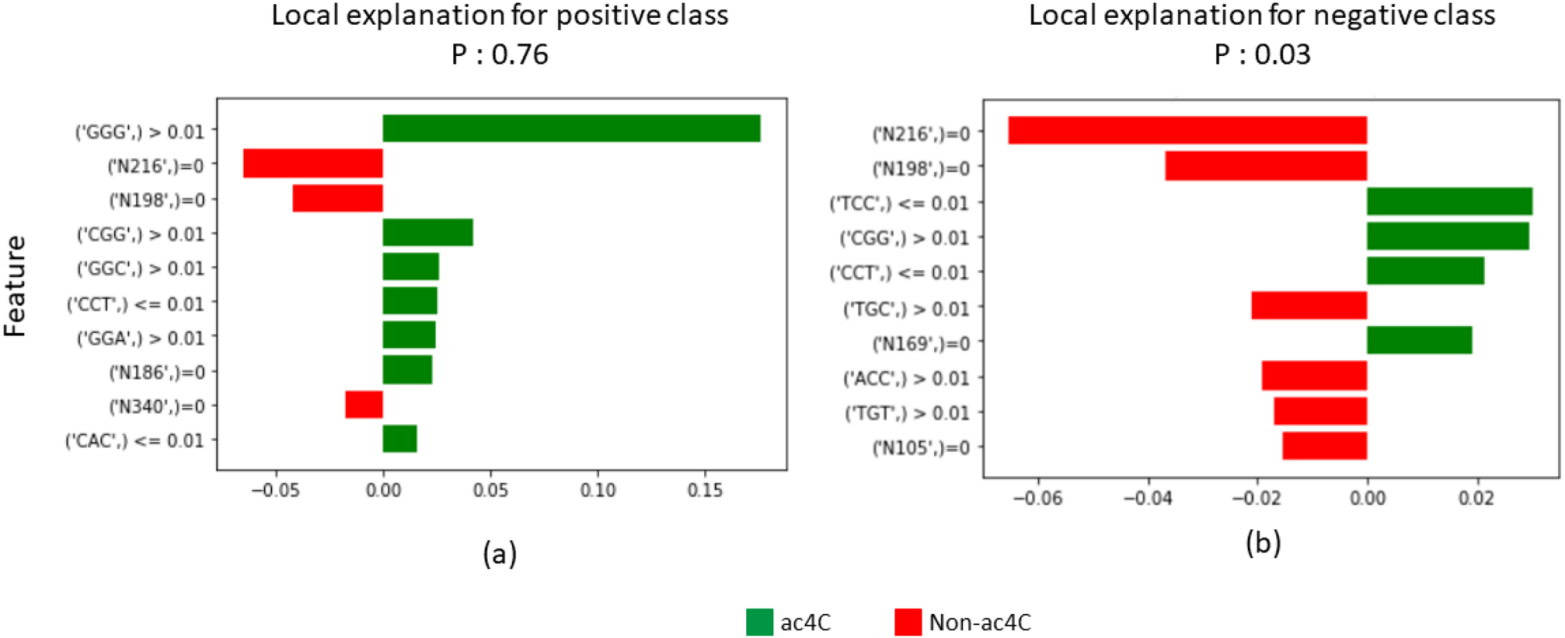
Local Interpretable Model-agnostic Explanations (LIME). The green bar shows the weighted features that support classification as ac4C; the red bars are the weighted features that oppose classification as ac4C. The LIME output of a positive sequence is shown in (**a**), while the LIME output of a negative sequence is shown in (**b**).

In this section, we discuss the contribution of each feature to the model’s outcome. We adopted two techniques, Shapley Additive Explanations (SHAP) and Local Interpretable Model-agnostic Explanations (LIME), to understand the importance and contribution of each feature^17–19^. SHAP utilizes local explanations and game theory, and is suitable for the interpretation of machine learning models. The XGboost classifier measures feature importance based on information gain, cover, or weight, whereas the SHAP value is a locally accurate additive method that indicates the importance of most global features for classification. The top 20 most important features of the trained models with both local and global EIIP and PseEIIP are shown in Fig. 4. The lower feature values are shown in blue, while the higher feature values are in red. The predicted ac4C sites are strongly related to higher frequencies of PseEIIP values of GGG, CGG, GGC, and CCC are rich nucleotides. On the other hand, the lower frequencies of EIIP at the non-enriched nucleotide positions N198 and N216 are associated with a lower predicted probability of the sequences being ac4C sites. To further understand the effects of these features on the prediction, we plot the LIME output for a positive sequence Fig. 5a and a negative sequence Fig. 5b. LIME provides more details than SHAP as it specifies a range of feature values that allow a given feature to exert its influence. In Figure 5, the green bars show the weighted features that support the classification of ac4C sites, while the red bars show the weighted features that support the classification of non-ac4C sites. These results agree with the SHAP results.

## Materials and methods

### Benchmark datasets

To develop a useful computational model, we obtained the benchmark datasets from PACES (http://www.rnanut.net/paces/)^6^. These datasets were originally extracted from 2134 genes prepared by Danial Arango et al.^5^ The positive and negative sequences have been experimentally validated as ac4C sites and non-ac4C sites, respectively. Each sequence in the positive and negative datasets has five consecutive CXX motifs in the center where $X\in \{A, C, G, T\}$. The length of the sequences in the benchmark datasets is 415 nt. The benchmark training dataset contains 1160 positive samples and 10855 negative samples. The independent testing dataset contains 469 positive samples and 4343 negative samples. Furthermore, we utilized fivefold cross-validation during the training process for quality control purposes. Thus, the training dataset was split into five folds, with each folds containing 232 positive samples and 2171 negative samples. Four folds were utilized for training and the remaining fold was utilized for testing. The training of the proposed model takes five sequential cycles; the final performance is the average of the results obtained from all five folds.

### Feature extraction

Feature extraction plays a key role in construction of reliable computational methods. In this study, we used the following five mRNA sequence extraction techniques to extract feature from mRNA sequences.

#### One-hot encoding

The input RNA sequence was encoded using the one-hot technique, in which A is encoded by (1,0,0,0), T is encoded by (0,1,0,0), G is encoded by (0,0,1,0) and C is encoded by (0,0,0,1). Thus, each input sequence in the benchmark dataset was encoded by a vector with a length of $415\times 4 =1660$.

#### Nucleotide chemical property (NCP)

The nucleotides of an mRNA sequence can be classified into three groups based on ring structure, functional groups, and hydrogen bonds. Several recent studies utilized chemical nucleotide properties for different problems^20–22^. Briefly, C and T have a single-ring structure, whereas A and G have two-ring structures; A and C belong to the amino group, while G and T belong to the keto group; and A and T form strong hydrogen bonds, whereas C and G form weak hydrogen bonds. According to the enumeration of these chemical properties, each mRNA sequence was encoded by a 3-dimensional vector (x, y, z), where x, y, and z are derived as follows:

1 $$\begin{aligned} x_i=\left\{ \begin{array}{ll} 1 \quad {if} \quad {n_i} \in \{A,C\}\\ 0 \qquad {other} \\ \end{array} \right. , y_i=\left\{ \begin{array}{ll} 1 \quad {if} \quad {n_i} \in \{A,G\}\\ 0 \qquad {other} \\ \end{array} \right. , z_i=\left\{ \begin{array}{ll} 1 \quad {if} \quad {n_i} \in \{A,T\}\\ 0 \qquad {other} \\ \end{array} \right. \end{aligned}$$

where $x_i$, $y_i$, and $z_i$ represent the NCP values of the nucleotide *n* at position *i*. Thus, each input sequence from the benchmark dataset was encoded by a vector with a length of $415 \times 3$ =1245.

#### Nucleotide density (ND)

Nucleotide density provides information about nucleotide frequency as well as nucleotide location information in an mRNA sequence. The ND has been utilized in various studies^20^. The ND $d_i$ of nucleotide $n_j$ as position j is expressed as:

2 $$\begin{aligned} d_{i}=\frac{ 1 }{|N_{i}|} \sum _{j=1}^{l} f(n_{j}) ; \quad {f(n_j)} = \left\{ \begin{array}{ll} 1 \quad {if} \quad {n_{j}} = p,\quad {p} \in \{A,C,G,T\}\\ 0 \qquad {otherwise} \\ \end{array} \right. \end{aligned}$$

where $N_i$ is the length of the i-th prefix subsequence from the first position to the *i*th position, l is the sequence length. Thus, each input sequence from the benchmark datasets was encoded by a vector with a length of 415. In general, we concatenate NCP with ND. Thus, the dimension of the resultant vector is 1245 + 415 = 1660.

#### K-mer

In this study, we also applied a widely used approach, K-mer, to represent the mRNA sequence. K-mer refers to the calculation of the frequencies of all possible sub-sequences of length k. It has been utilized for various problems^23,24^. In this paper, we used k = 1, 2, and 3 where 1-mer represents single-nucleotide (SN), 2-mer represents di-nucleotide (DN), and 3-mer represents tri-nucleotide (TN). Thus, each input sequence from the benchmark datasets was encoded by a vector with a length of 4 + 16 + 64 = 84.

#### EIIP+PseEIIP

The EIIP values of the nucleotides were proposed by Nair and Sreenadhan^25^, and have been utilized to address various problems in the field of bioinformatics^26,27^. In EIIP, each nucleotide of an mRNA sequence is encoded by a numerical value corresponding to the distribution of free electron energies. A is encoded by 0.1260, C is encoded by 0.1340, G is encoded by 0.0806, and T is encoded by 0.1335. Furthermore, pseudo-EIIP (PseEIIP) is applied to tri-nucleotides of the mRNA sequence by taking the mean EIIP value of each nucleotide. The mRNA sequence is encoded using PseEIIP by a vector of length 64 as:

3 $$\begin{aligned} PseEIIP = [EIIP_{AAA}.f_{AAA},EIIP_{AAC}.f_{AAC},\ldots ,EIIP_{TTT}.f_{TTT}] \end{aligned}$$

where $f_{xyz}$ is the normalized frequency of $i^{th}$ trinucleotide, $\hbox {EIIP}_{{xyz}}$ = EIIPx+EIIPy+EIIPz, and x, y, z $\in \{A, C, G, T\}$. The resulting dimension of the PseEIIP feature vector is 64. Hence, each input sequence from the benchmark dataset was encoded by a vector with a length of 415 + 64 = 479. The 415-dimension vector represents the EIIP values of the input sequence and the 64-dimension vector represents the PseEIIP values of the input sequence.

### XGBoost classifier

eXtreme Gradient boost (XGboost) is one of the most reliable machine learning classifiers, and has been widely applied to bioinformatics problems^28,29^. It is based on a tree model that utilizes a boosting algorithm for classification. To reduce the complexity of the model and control overfitting, regularization items are added to the cost function. Furthermore, the parallel computing function is supported by the XGboost algorithm, which improves computational speed. On the other hand, it is a highly flexible system in which the optimization goals and evaluation criteria can be customized by the user. Moreover, XGboost handles imbalanced datasets easily. Therefore, we proposed using the XGboost algorithm to solve the classification problem related to imbalanced datasets. We applied the grid search method to identify the optimal hyperparameters in XGboost. The optimal hyperparameter values are shown in Table 3.

**Table 3:** The optimal hyper-parameter values of the proposed model, XG-ac4C.

The hyper-parameter	The optimal value
N-estimators	1200
Learning-rate	0.01
Min-child-wieght	5
Max-depth	5
Colsample-bytree	0.8
Gamma	5
Subsample	0.8
Scale-pos-weight	6

### Evaluation metrics

In this work, we evaluate the proposed model using the area under the receiver operating characteristic curve (ROC) and the area under the precision-recall curve (PRC). Because the benchmark datasets are imbalanced, PRC is the best choice for studying the performance of the proposed model^12^. Moreover, the accuracy (ACC), specificity (Sp), sensitivity (Sn), and Matthews correlation coefficient (MCC) were utilized in various recent published studies to evaluate classifier quality in the field of bioinformatics^30–37^. Thus, we also use them to evaluate the performance of the proposed model. These evaluation metrics are defined as:

4 $$\begin{aligned} {{\,\mathrm{ACC}\,}}= & {} 1-(\frac{N_-^+ + N_+^-}{N^+ + N^-}) \end{aligned}$$

5 $$\begin{aligned} {{\,\mathrm{SN}\,}}= & {} 1-(\frac{N_-^+ }{N^+ }) \end{aligned}$$

6 $$\begin{aligned} {{\,\mathrm{SP}\,}}= & {} 1-(\frac{N_+^- }{N^- }) \end{aligned}$$

7 $$\begin{aligned} {{\,\mathrm{MCC}\,}}= & {} \frac{1-(\frac{N_-^+ + N_+^-}{N^+ + N^-})}{\sqrt{(1+\frac{N_+^- - N_-^+}{N^+ })(1+\frac{N_-^+ - N_+^-}{N^- })}} \end{aligned}$$

where $N^+$ represents the acetylcytidine sties, non-acetylcytidine sites are represented by ${N^-}$. ${N_+^-}$ represents the acetylcytidine sites incorrectly identified as non-acetylcytidine, and ${N_-^+}$ represents the number of non-acetylcytidine sites that are incorrectly classified as acetylcytidine sties.

## Web-server

**Figure 6:**
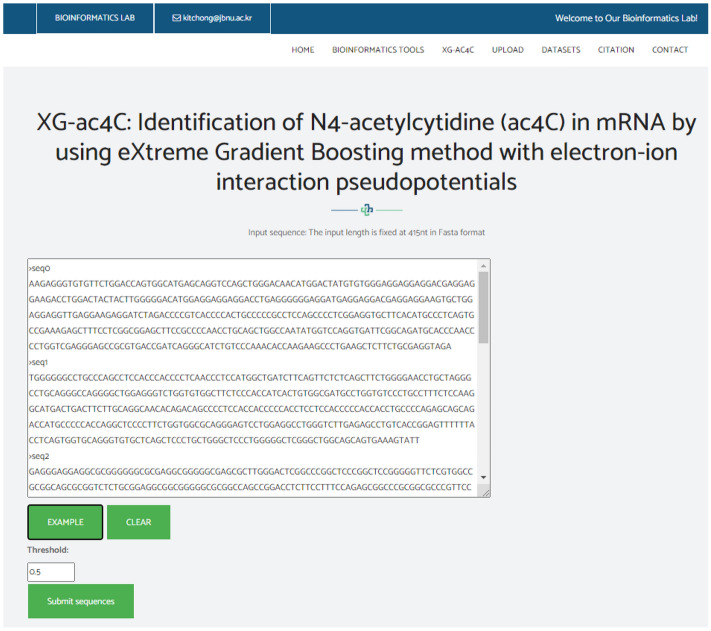
The web server window in which a user can paste an mRNA sequence in Fasta format for the prediction of ac4C sites.

**Figure 7:**
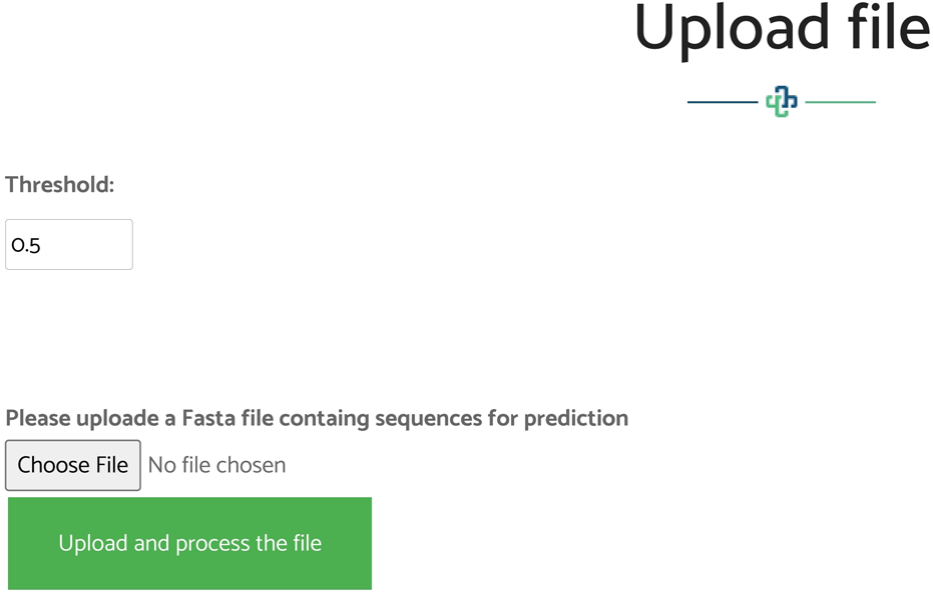
The web server window in which a user can upload an mRNA sequence in a Fasta file.

We established a user-friendly and freely accessible web server for the proposed method to facilitate future research. The established web server supports classification of ac4C sites using either direct sequences in Fasta format, as shown in Fig. 6, or direct upload of a Fasta file, as shown in Fig. 7. The web server was developed using the Python programming language with the Flask library. It is available at http://nsclbio.jbnu.ac.kr/tools/xgac4c/.

## Conclusion

Accurate identification of mRNA post-transcriptional modifications, such as acetylcytidine (ac4C), is crucial to furthering our understanding of various biological mechanisms. In this work, we developed an efficient and robust machine learning model that identifies acetylated mRNA sites. Moreover, the proposed model utilizes EIIP features to accurately predict ac4C sites. The proposed model, XG-ac4C, outperforms state-of-the-art methods on both cross-validation and independent tests. In addition, we visualized feature importance in XG-ac4C using the SHAP and LIME explainer techniques. Finally, the XG-ac4C model can be used to facilitate many areas of biological research; thus, we developed a freely accessible web server which can be found at http://nsclbio.jbnu.ac.kr/tools/xgac4c/.

## Supplementary information

Supplementary information.
