# Supplementary material for: XG-ac4C: identification of N4-acetylcytidine (ac4C) in mRNA using eXtreme gradient boosting with electron-ion interaction pseudopotentials
Source: Sci Rep. 2020 Dec 1;10:20942. doi: 10.1038/s41598-020-77824-2 (PMC7708984; doi:10.1038/s41598-020-77824-2)
Supplement: Supplementary file 1 — Supplementary information. [file 41598_2020_77824_MOESM1_ESM.pdf]

XG-ac4C: Identification of N4-acetylcytidine (ac4C) in mRNA using eXtreme Gradient Boosting with electron-ion interaction pseudopotentials

Waleed Alam[1], Hilal Tayara[2,*], and Kil To Chong[1,3,]**

[1]Department of Electronics and Information Engineering, Jeonbuk National University, Jeonju, 54896, South Korea
[2]School of International Engineering and Science, Jeonbuk National University, Jeonju, 54896, South Korea
[3]Advanced Electronics and Information Research Center, Jeonju, 54896, South Korea
[*]hilaltayara@jbnu.ac.kr
[**]kitchong@jbnu.ac.kr

Figure 1: The ROC and PRC of the proposed model on different encoding technique.

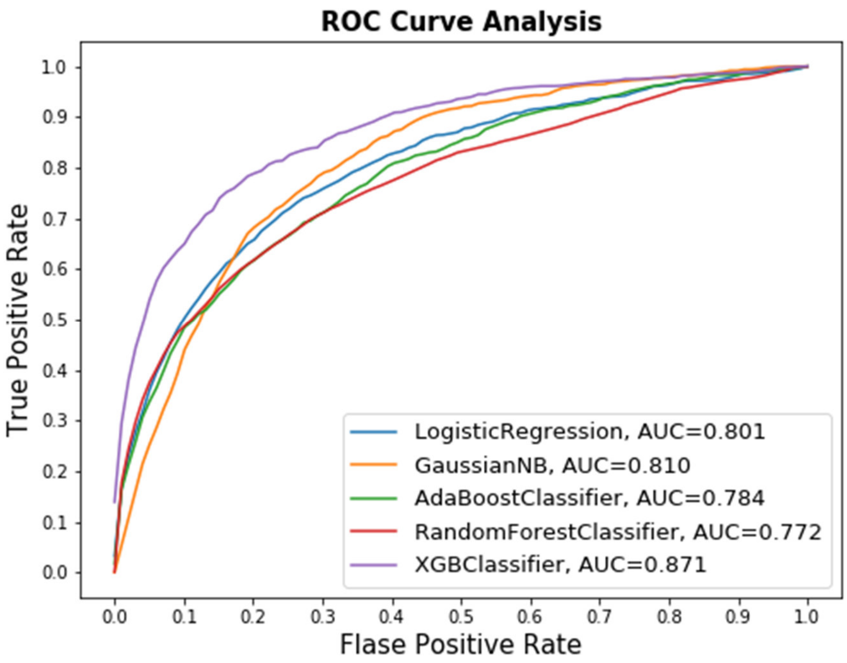

(a1) Cross-validation ROC of One-hot encoding

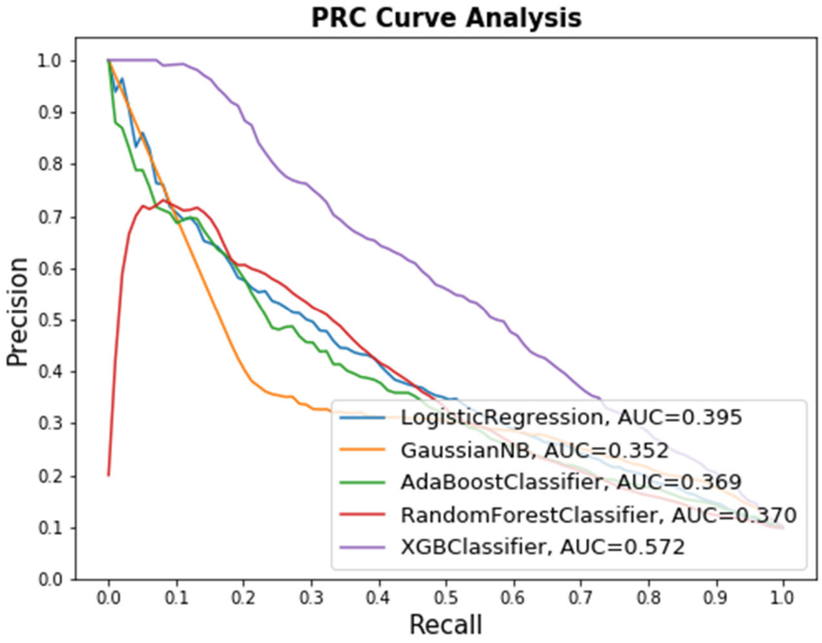

(b1) Cross-validation PRC of One-hot encoding

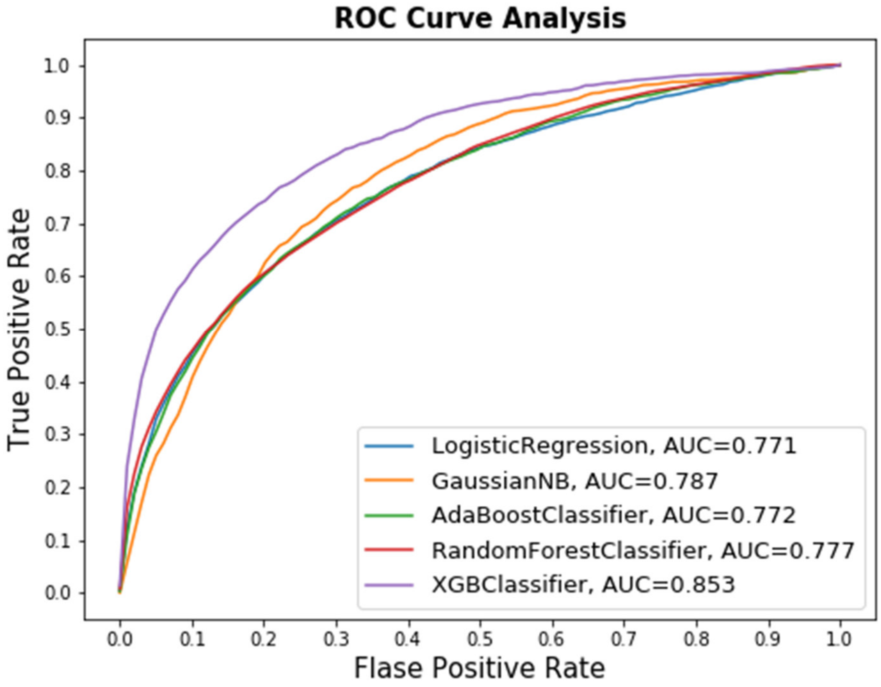

(c1) Independent test ROC of One-hot encoding

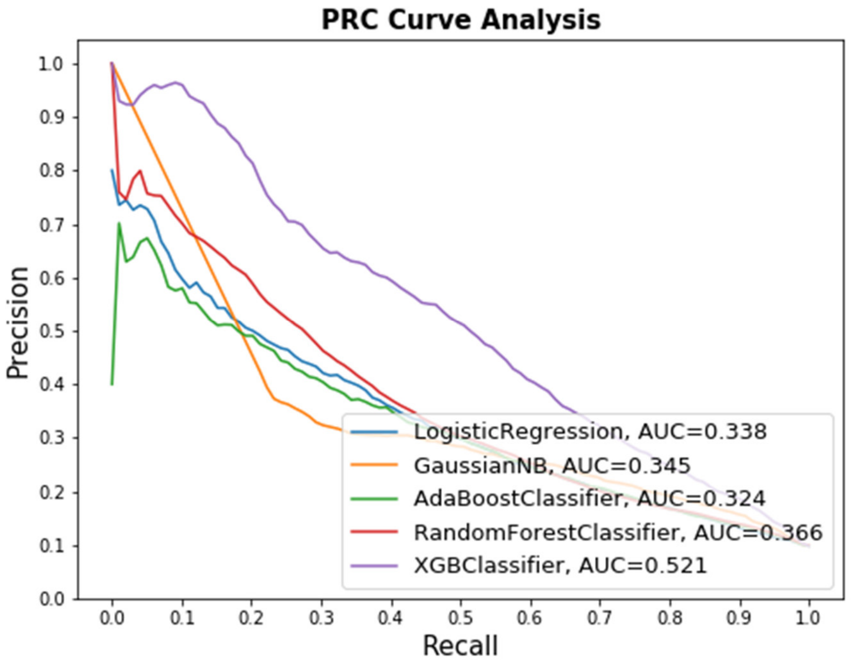

(d1) Independent test PRC of One-hot encoding

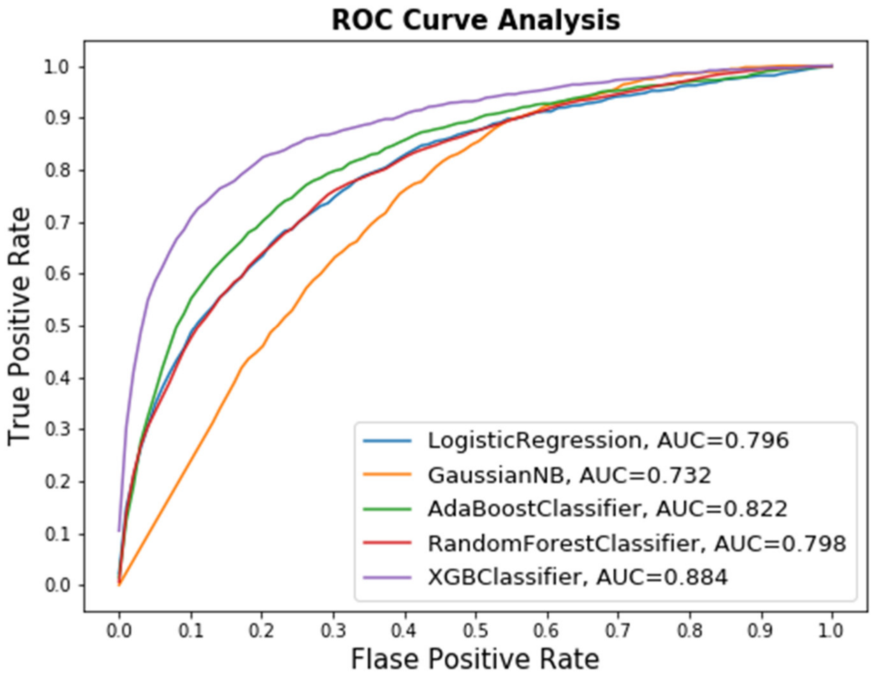

(a2) Cross-validation ROC of NCP-ND encoding

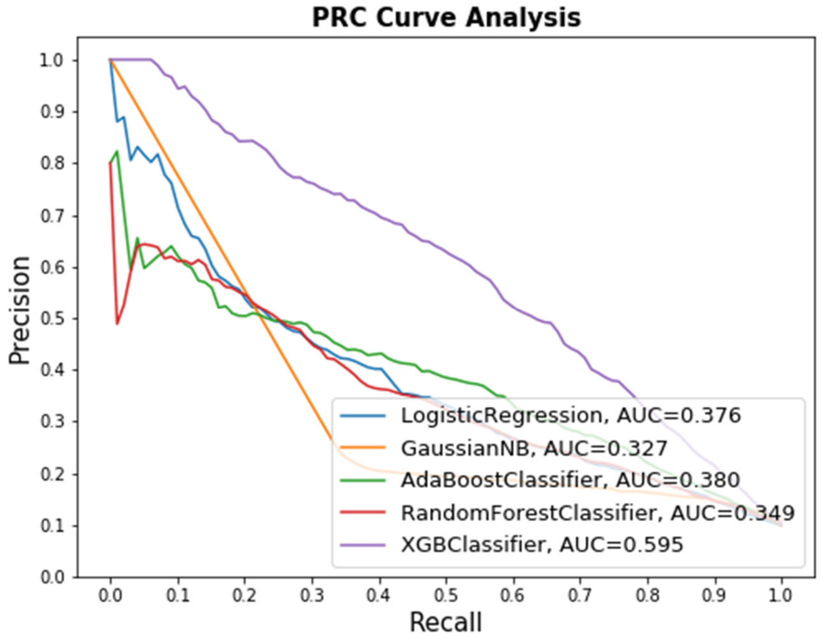

(b2) Cross-validation PRC of NCP-ND encoding

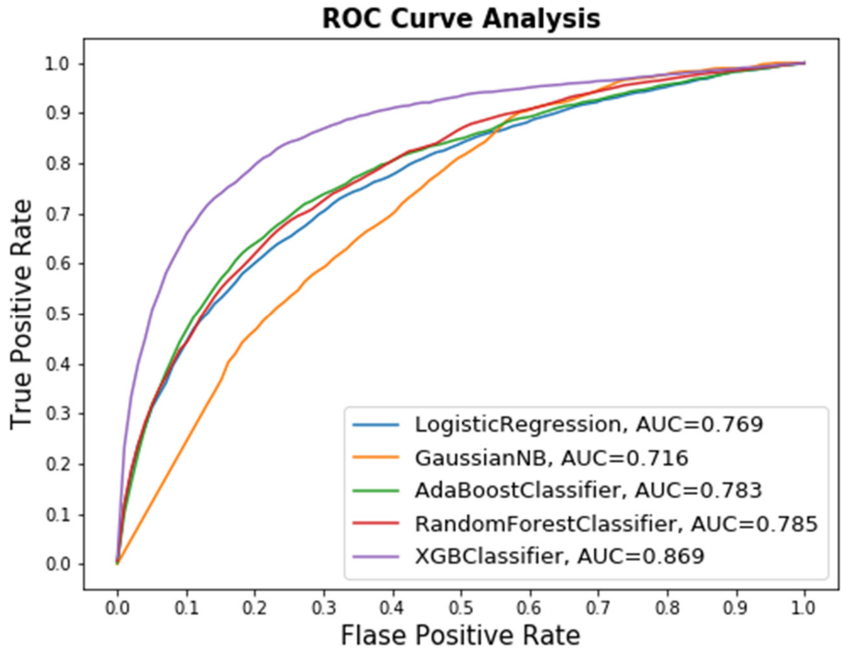

(c2) Independent test ROC of NCP-ND encoding

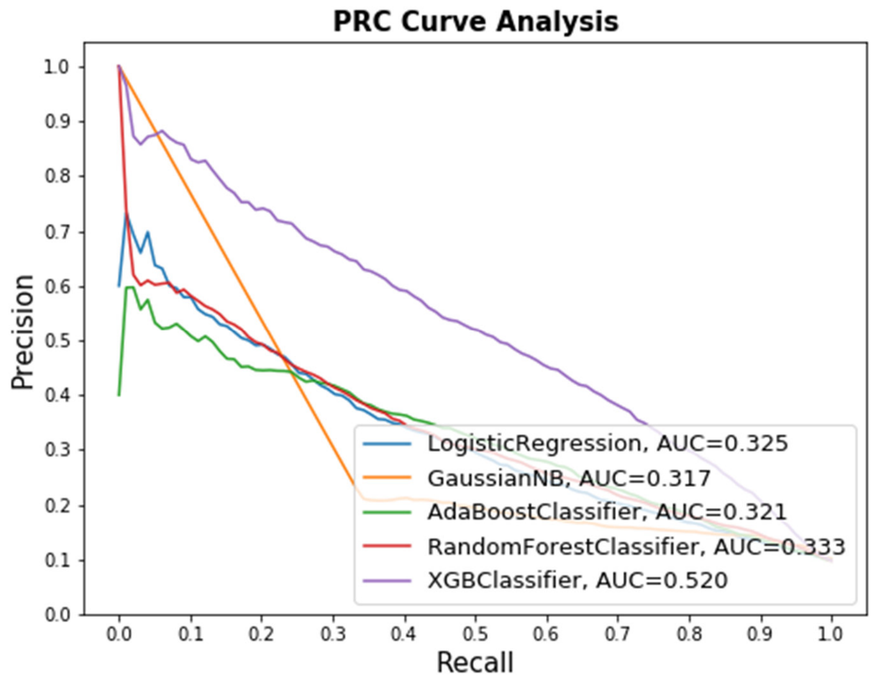

(d2) Independent test PRC of NCP-ND encoding

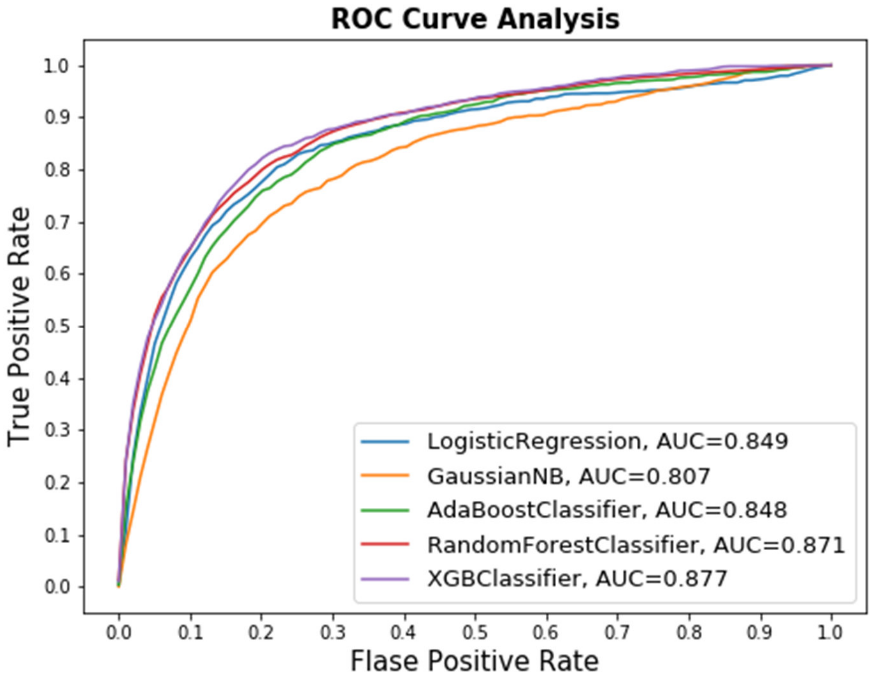

(a3) Cross-validation ROC of K-mer encoding

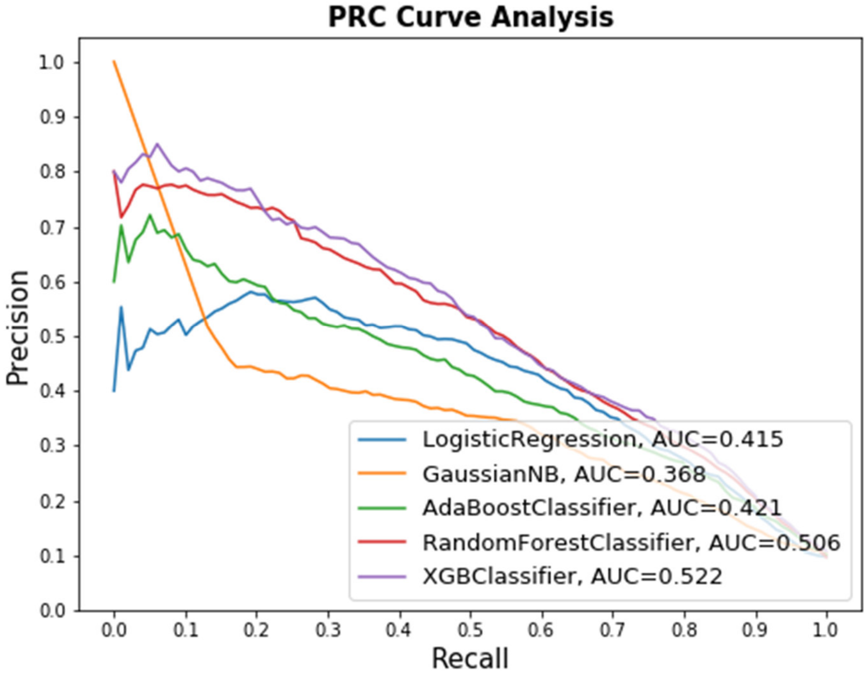

(b3) Cross-validation PRC of K-mer encoding

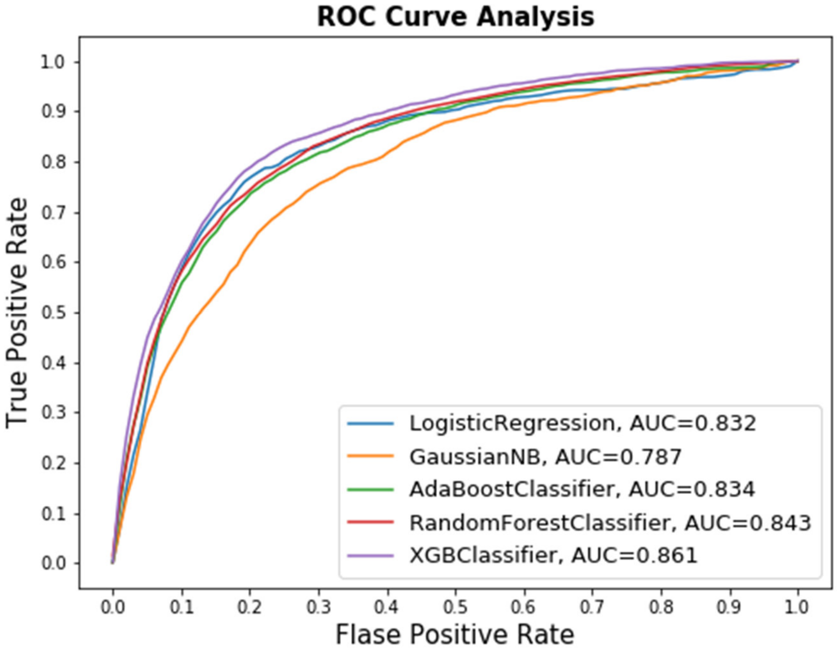

(c3) Independent test ROC of K-mer encoding

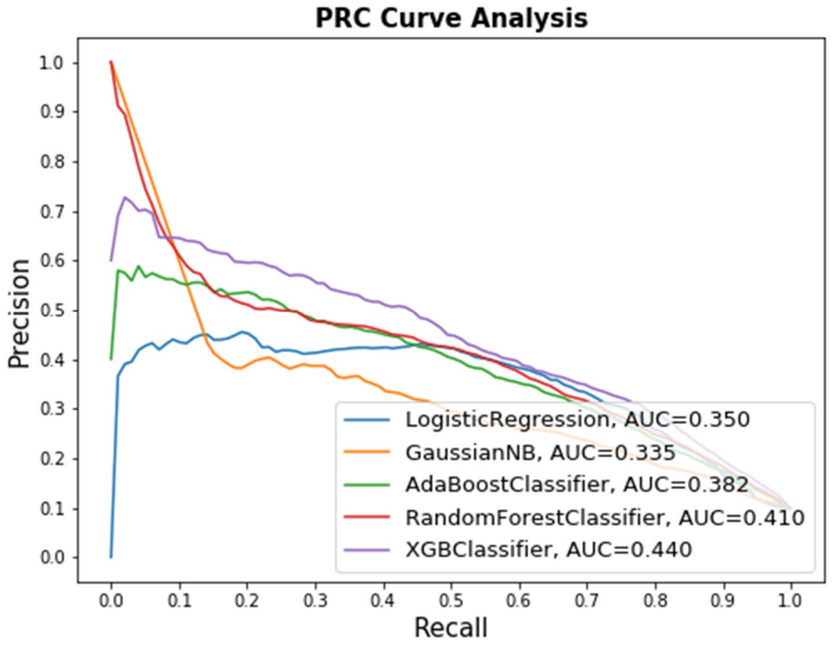

(d3) Independent test PRC of K-mer encoding

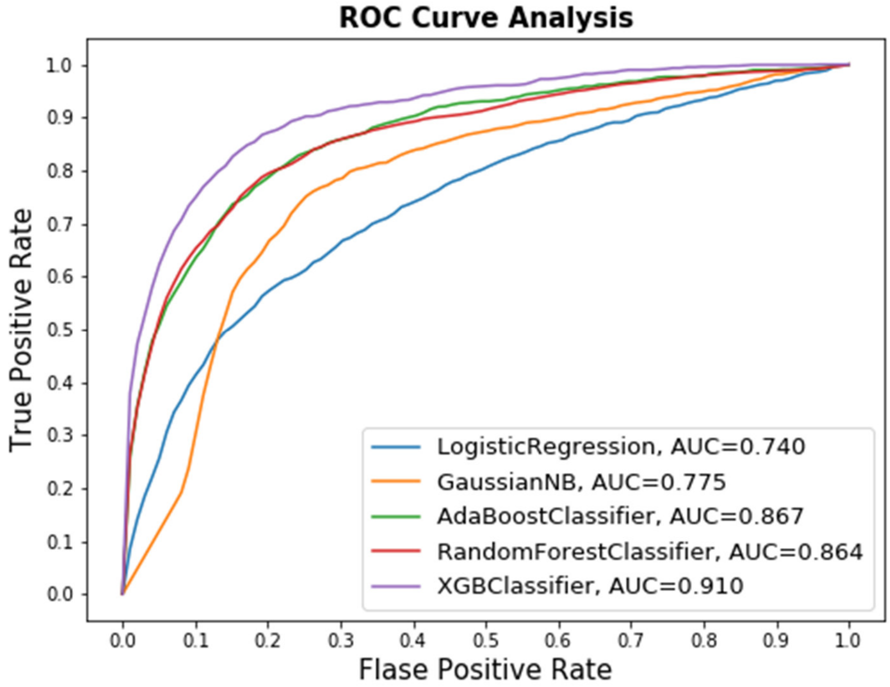

(a4) Cross-validation ROC of EIIP-PseEIIP encoding

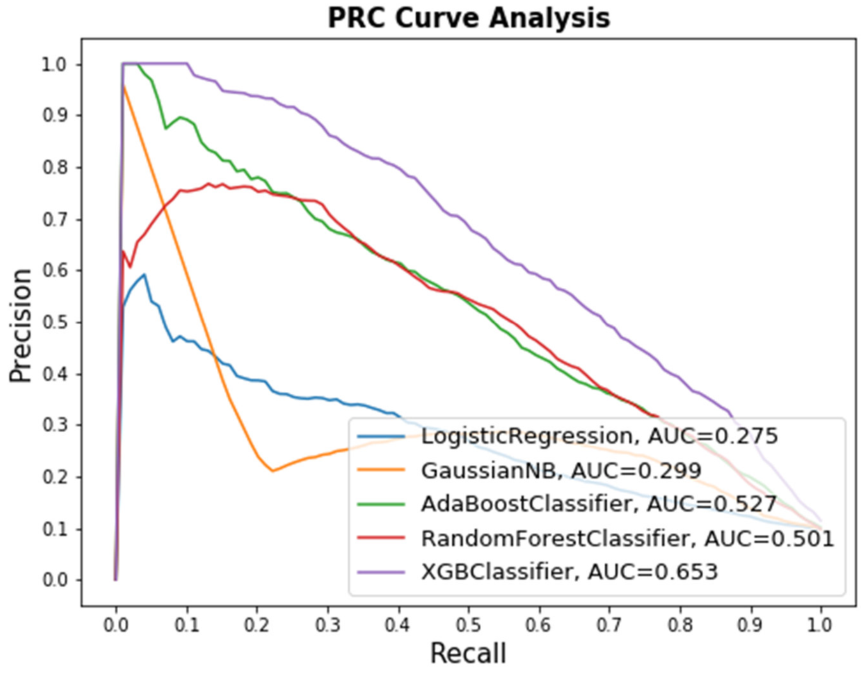

(b4) Cross-validation PRC of EIIP-PseEIIP encoding

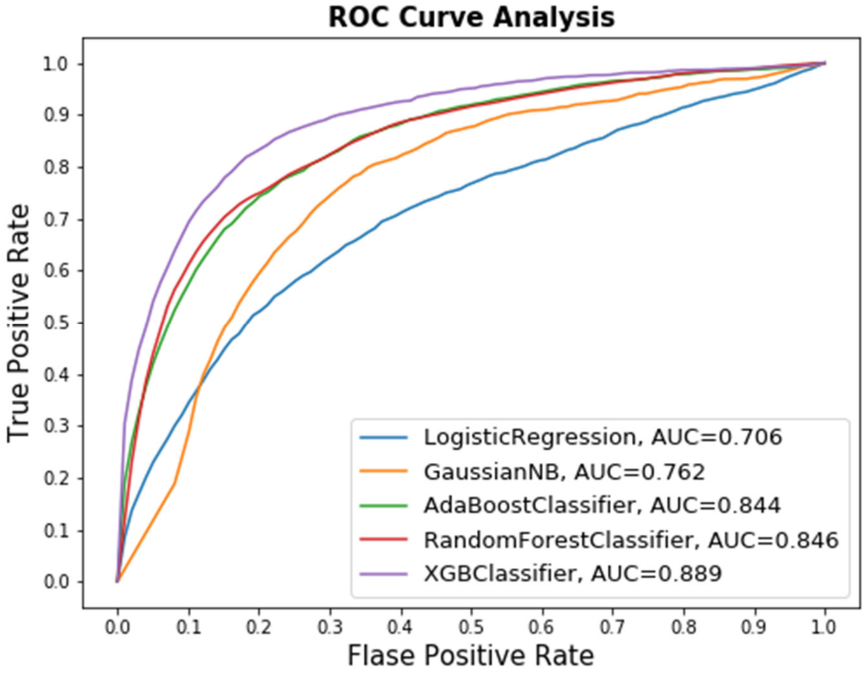

(c4) Independent test ROC of EIIP-PseEIIP encoding

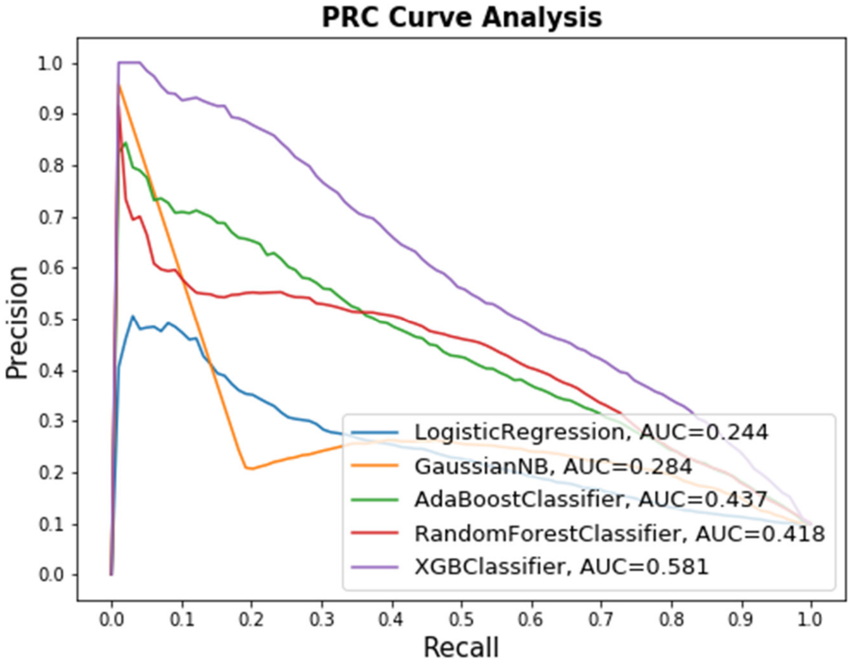

(d4) Independent test PRC of EIIP-PseEIIP encoding
